# Serum klotho associated with thyroid hormone in adults: A population-based cross-sectional research

**DOI:** 10.1371/journal.pone.0301484

**Published:** 2024-05-02

**Authors:** Xia Zhang, Xuekui Liu, Lin Li, Yan Zhang, Qing Li, Houfa Geng, Li Shi, Ben Wang, Qinqin Qiu, Tianpei Yu, Yiquan Sang, Liying Wang, Wei Xu, Jun Liang

**Affiliations:** 1 The Xuzhou Clinical College of Xuzhou Medical University, Xuzhou, Jiangsu, China; 2 Department of Endocrinology, Xuzhou Central Hospital, Xuzhou Institute of Medical Sciences, Xuzhou Clinical School of Nanjing Medical University, Affiliated Hospital of Medical School of Southeast University, Jiangsu, China; 3 Bengbu Medical College, Bengbu, Anhui, China; Wroclaw University of Science and Technology: Politechnika Wroclawska, POLAND

## Abstract

**Background and study aim:**

The klotho protein, a multifunctional protein, has been shown to be associated with a wide range of endocrine diseases and has been linked to thyroid tumourigenesis. However, the relationship between serum klotho levels and thyroid hormones remains poorly understood. This study aimed to explore the correlation between serum klotho levels and thyroid hormones.

**Methods:**

Data was obtained from the NHANES cycles 2007–2008, 2009–2010, and 2011–2012. A total of 4674 participants were recruited for this study. Statistical analysis was using multiple linear regression analyses, and restricted cubic spline plots (RCS) to investigate the association between serum klotho levels and serum levels of thyroid hormones.

**Results:**

In the unadjusted covariate model, ln(klotho) significantly positively correlated with tT3, tT4, fT3, tT4/fT4, and tT3/fT3 (all P<0.01) and negatively correlated with TSH, tT4/tT3, and fT4/fT3 (all P<0.05). Furthermore, tT3, tT4, fT3and tT3/fT3 (P < 0.05) were still significant in the adjusted model. And it is worth noting that there is an approximately L-shaped nonlinear relationship between ln(klotho) and fT3,tT3 with a cut-off point of 6.697 (P-non-linear < 0.05). The stratification analysis showed gender and iodine level differences in the relationship between serum Klotho levels and thyroid hormones.

**Conclusion:**

There is an L-shaped nonlinear relationship between ln(klotho) and fT3, tT3, suggesting that klotho could be involved in the physiological regulation of thyroid function.

## Introduction

The thyroid gland is one of the largest endocrine glands in the body and produces thyroid hormones, namely thyroxine (T4) and triiodothyronine (T3) [1]. Thyroid hormones act on almost all nucleated cells and play vital roles in the body [2,3]. The regulation of thyroid hormones is associated with a variety of substances such as sodium/iodide isotransporter protein (NIS), thyroglobulin (TG), thyroid peroxidase (TPO), monoiodotyrosine (MIT), diiodotyrosine (DIT) and μ-Crystallin (CRYM) [4–8]. New studies have found that klotho protein is associated with the development of thyroid tumours [9]. However, the association between klotho protein and thyroid hormones has not been investigated.

The klotho protein was first identified by Kuro-o et al. in 1997 [10]. The klotho gene encodes a 130 kDa type I single-pass transmembrane glycoprotein called α-Klotho, which has three isoforms (α, β, and γ) [11,12]. Klotho proteins have been shown to exert anti-oxidative, anti-inflammatory, and anti-apoptosis effects and regulate calcium and phosphorus metabolism [13]. Pawlikowski et al. reported that a low expression of a-Klotho was involved in thyroid tumor formation [14]. Previous study has demonstrated that fT3 levels may be positively correlated with sKlotho concentrations [15]. In 3T3-L1 adipocytes, T3 significantly increased the expression levels of membrane form of the klotho gene [16]. Based on the above studies, it is suggested that klotho protein is associated with thyroid cancer and fT3, however, the correlation between klotho protein and thyroid hormones and exactly how it is associated has not been clarified.

This study used serum klotho and thyroid function data for participants who participated in the National Health and Nutrition Examination Survey (NHANES) cycles 2007–2008, 2009–2010, and 2011–2012. This study explored the association between serum klotho and thyroid function using data from NHANES.

## Methods

### Study population

**Fig 1:**
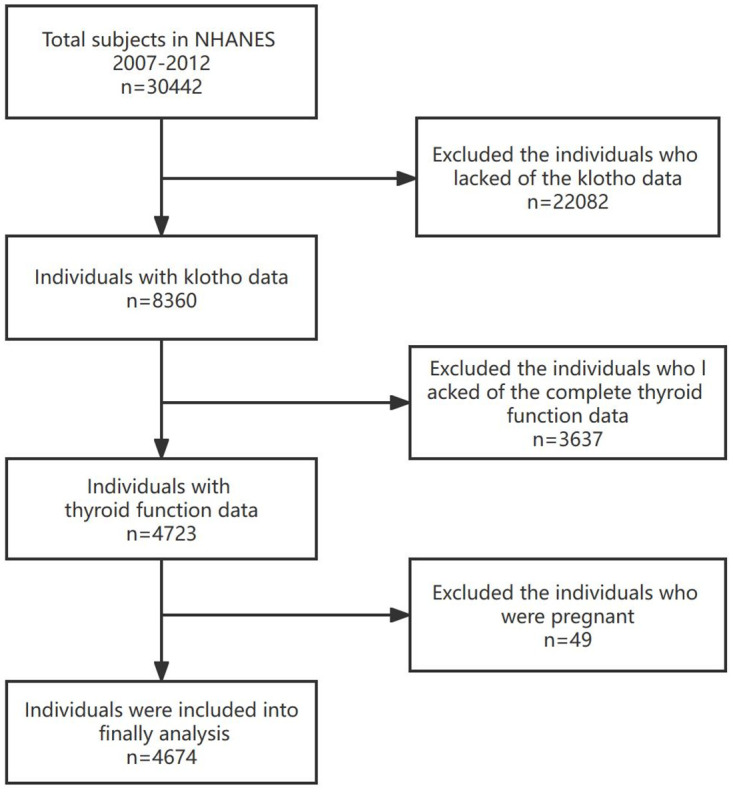
Flowchart of the study population for natriuresis.

The National Health and Nutrition Examination Survey (NHANES) is an ongoing nationwide survey conducted every two years to evaluate the health and nutritional status of the non-institutionalized U.S. population. NHANES uses sophisticated multi-stage probability sampling to select participants representing the non-institutionalized civilians in the U.S. Detailed information on survey design and methods was as previously described [17]. The NHANES study protocols were approved by the National Center for Health Statistics (NCHS) Research Ethics Review Committee. To ensure the protection of the participants’ rights, NHANES has obtained informed written consent from all the individuals involved in the study. A total of 8360 subjects were selected from the NHANES cycles 2007–2008, 2009–2010, and 2011–2012. However, 3637 subjects were excluded due to missing thyroid function test results, and 49 subjects were excluded due to pregnancy. Therefore, 4674 eligible subjects were included in the analysis. A flow chart showing the screening of the study participants is shown in Fig 1.

### Measurement of serum thyroid hormones

Blood samples were collected into 15 ml vacutainers, then centrifuged to obtain serum for further biochemical analysis. The total serum T4 (TT4), T3 (TT3), and free T3 (FT3) levels were determined using the competitive binding assay method. Free T4 (FT4) serum levels were determined using a two-step enzyme immunoassay method. The serum thyroid stimulating hormone (TSH) was measured using the third- and second-generation immune enzymatic (sandwich) assay.

### Determination of serum klotho levels

Soluble-Klotho acts as an endocrine or paracrine factor for various target organs. The Northwest Lipid Metabolism and Diabetes Research Laboratory, affiliated with the University of Washington, measured the serum klotho levels in whole blood samples using ELISA kits. Prior to the analysis, all samples were frozen at 80°C. Two parallel holes were made in the ELISA plate to measure the klotho levels of the quality control samples, and the average value was considered as the final concentration. For healthy people, the reference range for serum Klotho levels are between 285.8 to 1638.6 pg/mL with a mean of 698.0 pg/mL.

### Statistical analysis

Due to skewness, serum levels of klotho were log-transformed to approximate a normal distribution. The ln(klotho) levels and baseline indicators were categorized by quartiles. Continuous variables were expressed as standard deviation ± mean or median (IQR), and categorical variables were shown as counts (%). Comparisons between groups were made using Pearson’s χ2 test for categorical variables and ANOVA and Kruskal-Wallis H-test for continuous variables. Use violin charts to visualize thyroid hormone levels more intuitively under different Klotho groups. Exploring the relationship between klotho and thyroid hormones using multivariate linear regression and multivariate logistic regression (positive outcomes with thyroid hormone levels higher than three-quarters of a digit). Regression analysis was performed using two types of model calibration. Model 1 was not adjusted for any covariates, while Model 2 was adjusted for all baseline covariates. The relationship between ln(klotho) and serum thyroid hormone levels was tested for non-linearity. Restrictive cubic spline graph (RCS) is used to explore the nonlinear relationship between the two, and to find the cutoff point. Subgroup analyses were performed to examine whether the association between serum klotho and thyroid hormones was altered by gender and urinary iodine status. All statistical analyses were performed using the R software (version 4.3.0, R statistical computing base) and IBM SPSS statistical software (version 23.0). Two-tailed P-values of less than 0.05 were regarded as statistically significant.

## Results

### Anthropometric and biochemical characteristics of the study participants

**Fig 2:**
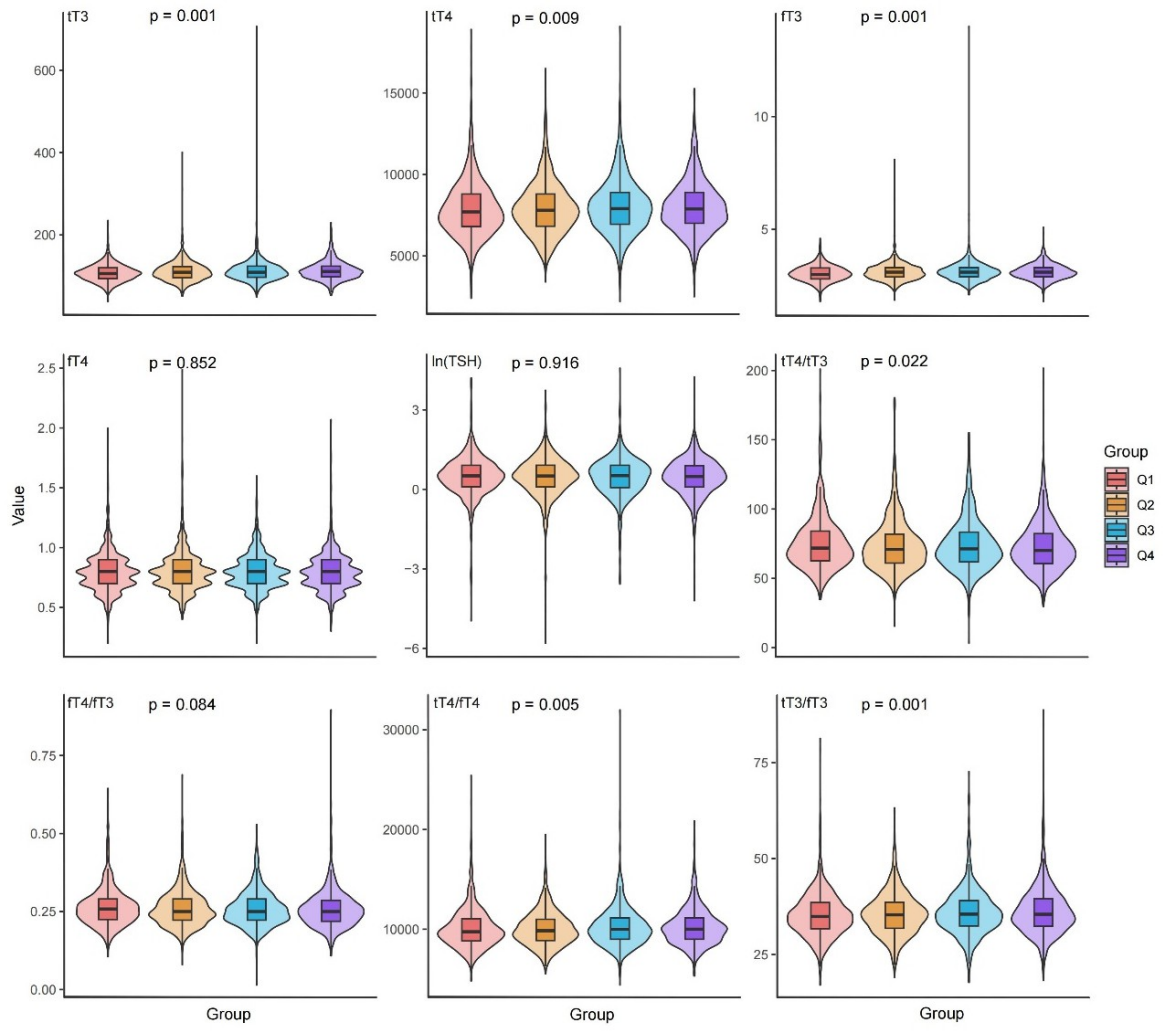
Violin plot of thyroid function(tT3, tT4, fT3, fT4, ln(TSH), tT4/tT3, fT4/fT3, tT4/fT4, tT3/fT3) under quartile grouping of ln (Klotho).

The baseline information of the study participants after stratification by ln(klotho) quartiles is shown in Table 1. A total of 4674 participants were included in the study after excluding participants who did not meet the eligibility criteria. The results showed that high tT3, tT4, fT3, tT4/fT4, and tT3/fT3 levels were associated with high serum levels of Klotho protein in females, non-Hispanic blacks, and patients with a positive history of smoking and alcohol use, the violin diagram further visualizes and demonstrates this point (Fig 2). However, high serum klotho levels were associated with lower values in height, weight, albumin, serum creatinine, glutathione, triglycerides, and uric acid levels. There was no significant difference between the groups in terms of diabetic patients, hypertensive patients, ghrelin, urinary iodine, urinary creatinine.

**Table 1:** Baseline characteristics of participants in the 2007–2012 continuous NHANES.

Characteristics	Q1[5.05,6.49] (n = 1168)	Q2[6.49,6.69] (n = 1169)	Q3[6.69,6.9] (n = 1168)	Q4[6.9,8.15] (n = 1169)	P-value
Sex					0.001
Female	621 (53.2%)	621 (53.1%)	558 (47.8%)	515 (44.1%)	
Male	547 (46.8%)	548 (46.9%)	610 (52.2%)	654 (55.9%)	
Age.years	60.000 (50.000 to 68.000)	59.000 (49.000 to 67.000)	58.000 (48.000 to 67.000)	56.000 (48.000 to 65.000)	0.001
Race					0.001
Mexican American	182 (15.6%)	174 (14.9%)	187 (16%)	187 (16%)	
Other Hispanic	114 (9.8%)	145 (12.4%)	130 (11.1%)	145 (12.4%)	
Non-Hispanic White	578 (49.5%)	579 (49.5%)	562 (48.1%)	476 (40.7%)	
Non-Hispanic Black	236 (20.2%)	198 (16.9%)	207 (17.7%)	294 (25.1%)	
Other Race	58 (5%)	73 (6.2%)	82 (7%)	67 (5.7%)	
Height.cm	167.800 (160.650 to 174.750)	167.150 (160.000 to 175.100)	166.100 (159.500 to 173.900)	165.200 (158.900 to 172.600)	0.001
Weight.kg	81.000 (69.300 to 93.800)	79.400 (68.200 to 94.400)	80.050 (68.350 to 93.450)	78.400 (67.400 to 91.400)	0.028
BMI	28.681 (25.137 to 32.730)	28.569 (25.107 to 32.534)	28.742 (25.200 to 32.804)	28.387 (24.914 to 33.019)	0.751
Smoke					0.001
None	524 (44.9%)	556 (47.6%)	582 (49.8%)	621 (53.1%)	
Yes	642 (55.1%)	613 (52.4%)	586 (50.2%)	548 (46.9%)	
Drink					0.001
None	279 (25.4%)	313 (28.7%)	332 (30.3%)	372 (34.4%)	
Yes	819 (74.6%)	778 (71.3%)	765 (69.7%)	709 (65.6%)	
Diabetes					0.612
None	938 (80.3%)	941 (80.7%)	939 (80.4%)	914 (78.3%)	
Yes	208 (17.8%)	195 (16.7%)	200 (17.1%)	223 (19.1%)	
Pre-diabetes	22 (1.9%)	30 (2.6%)	29 (2.5%)	31 (2.7%)	
High blood pressure					0.076
None	585 (50.2%)	631 (54.1%)	633 (54.3%)	644 (55.1%)	
Yes	581 (49.8%)	536 (45.9%)	533 (45.7%)	524 (44.9%)	
ALB.g/L.	42.000 (40.000 to 44.000)	43.000 (40.000 to 44.000)	42.000 (40.000 to 44.000)	42.000 (40.000 to 44.000)	0.011
ALT.U/L.	21.000 (17.000 to 27.000)	22.000 (17.000 to 29.000)	22.000 (17.000 to 29.000)	22.000 (17.000 to 30.000)	0.054
AST.U/L.	24.000 (20.000 to 27.000)	24.000 (21.000 to 29.000)	24.000 (20.000 to 29.000)	24.000 (20.000 to 29.000)	0.032
SCr.umol/L	80.440 (65.420 to 95.470)	77.790 (64.530 to 90.170)	74.260 (63.650 to 88.400)	72.490 (63.650 to 85.750)	0.001
Triglycerides.mmol/L	1.654 (1.073 to 2.495)	1.581 (1.084 to 2.337)	1.530 (1.039 to 2.371)	1.479 (0.960 to 2.281)	0.002
Uric acid.umol/L	339.000 (285.500 to 404.500)	333.100 (279.600 to 392.600)	327.100 (273.600 to 380.700)	309.300 (255.800 to 368.800)	0.001
Urine iodine.ug/L	157.850 (84.450 to 268.550)	147.600 (83.600 to 254.500)	153.300 (90.400 to 256.700)	147.600 (83.300 to 264.800)	0.498
Urine creatinine.mg/dl	101.000 (55.000 to 153.000)	103.000 (58.500 to 153.000)	104.000 (61.000 to 156.000)	100.000 (59.000 to 152.000)	0.602
Klotho.pg/ml	554.400 (488.450 to 613.400)	731.500 (694.300 to 766.400)	893.800 (847.800 to 938.500)	1168.000 (1071.600 to 1350.500)	0.001
tT3.ng/dL	106.000 (93.500 to 120.000)	109.000 (96.000 to 123.000)	109.000 (97.000 to 124.000)	111.000 (98.000 to 124.000)	0.001
tT4.ng/dL	7700.000 (6800.000 to 8800.000)	7800.000 (6820.000 to 8800.000)	7900.000 (6940.000 to 8900.000)	7880.000 (7000.000 to 8900.000)	0.009
fT3.ng/dL	3.000 (2.800 to 3.290)	3.100 (2.890 to 3.300)	3.100 (2.900 to 3.300)	3.100 (2.900 to 3.300)	0.001
fT4.ng/dL	0.800 (0.700 to 0.900)	0.800 (0.700 to 0.900)	0.800 (0.700 to 0.900)	0.800 (0.700 to 0.900)	0.852
TSH.uIU/mL	1.668 (1.103 to 2.474)	1.660 (1.095 to 2.480)	1.679 (1.067 to 2.478)	1.622 (1.092 to 2.450)	0.916
tT4/tT3	71.818 (62.548 to 84.137)	70.833 (61.029 to 81.897)	71.287 (61.838 to 83.406)	70.101 (60.656 to 82.243)	0.022
fT4/fT3	0.258 (0.224 to 0.290)	0.250 (0.222 to 0.290)	0.250 (0.222 to 0.290)	0.250 (0.219 to 0.286)	0.084
tT4/fT4	9750.000 (8828.431 to 11062.071)	9857.143 (8875.000 to 11000.000)	10000.000 (9000.000 to 11142.857)	10010.989 (9000.000 to 11142.857)	0.005
tT3/fT3	34.895 (31.702 to 38.545)	35.333 (31.818 to 38.611)	35.517 (32.424 to 39.047)	35.484 (32.368 to 39.535)	0.001

BMI, body mass index; ALB, albumin; AST, aspartate transaminase; ALT, alanine amiotransferase; fT4, free thyroxine; tT4, total thyroxine; fT3, free triiodothyronine; tT3, total triiodothyronine; TSH, thyroid-stimulating hormone; Q1,Q2,Q3,Q4, quartile grouping of ln(Klotho). The bold values indicate statistically significant differences.

### Association between klotho protein and serum thyroid hormones

The relationship between ln(klotho) as well as quartile grouping of ln(klotho) and thyroid hormones is shown in Table 2. In the unadjusted model, ln(klotho) was significantly positively correlated with tT3, tT4, fT3, tT4/fT4, tT3/fT3 and negatively correlated with TSH, tT4/tT3, fT4/fT3. However, after adjusting for all baseline covariates, ln(klotho) was significantly correlated with tT3 (β = 4.167, 95% CI: 1.947. 6.388), tT4 (β = 181.335, 95% CI: 32.251, 330.419), fT3 (β = 0.054, 95% CI: 0.019, 0.089), and tT3/fT3 (β = 0.729, 95% CI: 0.183, 1.275). In addition, Q2, Q3, and Q4 were more significantly positively correlated with tT3 and fT3 levels compared with Q1. While, only the Q3 group showed a significantly positive correlation with tT4 compared with Q1 (β = 188.280, 95% CI: 50.566, 325.995). For the tT3/fT3, only the Q4 group showed a significantly positive correlation compared with Q1 (β = 0.696, 95% CI:0.185, 1.207).

**Table 2:** Linear regression results between ln(Klotho) and its quartile with serum thyroid hormone.

		Model 1		Model 2	
		β (95% CI)	P-value	β (95% CI)	P-value
tT3	ln(Klotho)	6.642 (4.555,8.729)	0.001	4.167 (1.947,6.388)	0.001
	Q1	Reference		Reference	
	Q2	3.681 (1.699,5.663)	0.001	2.759 (0.711,4.807)	0.008
	Q3	4.901 (2.918,6.883)	0.001	3.694 (1.643,5.744)	0.001
	Q4	5.847 (3.865,7.829)	0.001	3.902 (1.826,5.978)	0.001
tT4	ln(Klotho)	206.820 (65.207,348.432)	0.004	181.335 (32.251,330.419)	0.017
	Q1	Reference		Reference	
	Q2	58.282 (-76.227,192.791)	0.396	97.801 (-39.769,235.372)	0.163
	Q3	166.515 (31.978,301.053)	0.015	188.280 (50.566,325.995)	0.007
	Q4	138.223 (3.714,272.732)	0.044	121.538 (-17.918,260.994)	0.088
fT3	ln(Klotho)	0.079 (0.045,0.113)	0.001	0.054 (0.019,0.089)	0.003
	Q1	Reference		Reference	
	Q2	0.070 (0.037,0.102)	0.001	0.047 (0.014,0.079)	0.005
	Q3	0.074 (0.042,0.107)	0.001	0.058 (0.025,0.090)	0.001
	Q4	0.072 (0.039,0.104)	0.001	0.049 (0.017,0.082)	0.003
fT4	ln(Klotho)	-0.001 (-0.014,0.013)	0.928	0.005 (-0.009,0.020)	0.477
	Q1	Reference		Reference	
	Q2	0.005 (-0.008,0.018)	0.464	0.006 (-0.008,0.019)	0.416
	Q3	0.002 (-0.011,0.015)	0.739	0.004 (-0.009,0.018)	0.539
	Q4	-0.004 (-0.016,0.009)	0.597	0.001 (-0.012,0.015)	0.861
TSH	ln(Klotho)	-0.267 (-0.523,-0.010)	0.042	-0.196 (-0.482,0.090)	0.179
	Q1	Reference		Reference	
	Q2	-0.127 (-0.371,0.117)	0.308	-0.100 (-0.363,0.164)	0.459
	Q3	-0.155 (-0.399,0.089)	0.213	-0.154 (-0.418,0.110)	0.254
	Q4	-0.184 (-0.428,0.060)	0.140	-0.104 (-0.371,0.163)	0.445
tT4/tT3	ln(Klotho)	-2.430 (-4.031,-0.829)	0.003	-0.826 (-2.417,0.764)	0.308
	Q1	Reference		Reference	
	Q2	-1.644 (-3.164,-0.123)	0.034	-0.573 (-2.041,0.894)	0.444
	Q3	-1.288 (-2.808,0.233)	0.097	-0.199 (-1.668,1.270)	0.790
	Q4	-2.468 (-3.988,-0.948)	0.001	-1.185 (-2.673,0.302)	0.118
fT4/fT3	ln(Klotho)	-0.007 (-0.012,-0.002)	0.01	-0.002 (-0.007,0.003)	0.416
	Q1	Reference		Reference	
	Q2	-0.004 (-0.009,0.001)	0.080	-0.002 (-0.006,0.003)	0.452
	Q3	-0.005 (-0.009,0.000)	0.057	-0.002 (-0.007,0.002)	0.334
	Q4	-0.007 (-0.012,-0.002)	0.004	-0.003 (-0.008,0.001)	0.170
tT4/fT4	ln(Klotho)	239.552(77.291,401.814)	0.004	130.947(-42.483,304.377)	0.139
	Q1	Reference		Reference	
	Q2	-12.842 (-166.918,141.233)	0.870	31.679 (-128.353,191.711)	0.698
	Q3	143.795 (-10.313,297.904)	0.067	146.239 (-13.960,306.438)	0.074
	Q4	193.520 (39.444,347.596)	0.014	111.786 (-50.438,274.010)	0.177
tT3/fT3	ln(Klotho)	1.231 (0.728,1.735)	0.001	0.729 (0.183,1.275)	0.009
	Q1	Reference		Reference	
	Q2	0.308 (-0.170,0.786)	0.207	0.270 (-0.234,0.774)	0.294
	Q3	0.643 (0.164,1.121)	0.008	0.447 (-0.057,0.952)	0.082
	Q4	1.070 (0.591,1.548)	0.001	0.696 (0.185,1.207)	0.008

fT4, free thyroxine; tT4, total thyroxine; fT3, free triiodothyronine; tT3, total triiodothyronine; TSH, thyroid-stimulating hormone; Q1,Q2,Q3,Q4, quartile grouping of ln(Klotho). The bold values indicate statistically significant differences.

Model 1(unadjusted) Covariates are not adjusted,Model 2(full-adjusted) Correct all covariates in the baseline table.

The levels of tT3, tT4, fT3, TSH, tT4/fT4, tT3/fT3, tT4/tT3, fT4/fT3 higher than their three-quarter quartile were used as a positive outcome and multivariate logistic regression was carried out, and the results are shown in Table 3. In the uncorrected model, klotho protein was a risk factor for elevated tT3, fT3, tT4/fT4, tT3/fT3 (P < 0.05), and after adequate correction of the model, klotho protein was still found to be a risk factor for elevated tT3, fT3, whereas its effect on tT4/fT4, tT3/fT3 was no longer significant.

**Table 3:** Logistic regression results between ln(Klotho) and its quartile with serum thyroid hormone.

		Model 1		Model 2	
		OR (95% CI)	P-value	OR (95% CI)	P-value
tT3	ln(Klotho)	1.545 (1.269,1.883)	<0.001	1.399 (1.127,1.737)	0.002
	Q1	Reference		Reference	
	Q2	1.420 (1.171,1.724)	<0.001	1.328 (1.082,1.631)	0.007
	Q3	1.453 (1.199,1.764)	<0.001	1.363 (1.110,1.674)	0.003
	Q4	1.562 (1.290,1.893)	<0.001	1.446 (1.178,1.778)	<0.001
tT4	ln(Klotho)	1.195 (0.981,1.455)	0.077	1.154 (0.933,1.427)	0.186
	Q1	Reference		Reference	
	Q2	1.008 (0.834,1.219)	0.932	1.067 (0.872,1.305)	0.530
	Q3	1.126 (0.934,1.357)	0.215	1.167 (0.956,1.426)	0.129
	Q4	1.080 (0.895,1.303)	0.422	1.057 (0.863,1.294)	0.594
fT3	ln(Klotho)	1.364 (1.130,1.647)	0.001	1.256 (1.016,1.555)	0.036
	Q1	Reference		Reference	
	Q2	1.370 (1.143,1.643)	0.001	1.241 (1.017,1.514)	0.034
	Q3	1.307 (1.089,1.569)	0.004	1.241 (1.017,1.516)	0.034
	Q4	1.375 (1.147,1.650)	0.001	1.262 (1.032,1.544)	0.023
fT4	ln(Klotho)	1.055 (0.871,1.278)	0.583	1.140 (0.920,1.413)	0.230
	Q1	Reference		Reference	
	Q2	0.920 (0.767,1.104)	0.371	0.921 (0.757,1.122)	0.415
	Q3	0.950 (0.792,1.139)	0.578	0.976 (0.802,1.187)	0.808
	Q4	0.974 (0.812,1.167)	0.772	1.027 (0.842,1.253)	0.790
TSH	ln(Klotho)	0.912 (0.748,1.111)	0.359	1.040 (0.835,1.294)	0.726
	Q1	Reference		Reference	
	Q2	1.003 (0.832,1.210)	0.972	1.000 (0.818,1.222)	0.997
	Q3	0.995 (0.826,1.200)	0.962	0.996 (0.814,1.218)	0.966
	Q4	0.967 (0.802,1.167)	0.729	1.096 (0.895,1.343)	0.374
tT4/tT3	ln(Klotho)	0.845 (0.693,1.029)	0.095	1.046 (0.833,1.313)	0.699
	Q1	Reference		Reference	
	Q2	0.830 (0.689,1.000)	0.050	0.975 (0.790,1.203)	0.811
	Q3	0.916 (0.761,1.101)	0.348	1.091 (0.887,1.343)	0.410
	Q4	0.826 (0.685,0.995)	0.045	0.973 (0.786,1.205)	0.802
fT4/fT3	ln(Klotho)	0.932 (0.765,1.136)	0.487	1.030 (0.820,1.294)	0.798
	Q1	Reference		Reference	
	Q2	0.972 (0.808,1.171)	0.767	1.061 (0.863,1.306)	0.573
	Q3	0.969 (0.805,1.167)	0.740	0.987 (0.801,1.217)	0.903
	Q4	0.854 (0.707,1.031)	0.100	0.905 (0.730,1.122)	0.365
tT4/fT4	ln(Klotho)	1.350 (1.109,1.645)	0.003	1.291 (1.047,1.592)	0.017
	Q1	Reference		Reference	
	Q2	1.239 (1.023,1.502)	0.029	1.196 (0.980,1.461)	0.078
	Q3	1.274 (1.052,1.543)	0.013	1.228 (1.007,1.500)	0.043
	Q4	1.431 (1.185,1.730)	<0.001	1.379 (1.131,1.683)	0.002
tT3/fT3	ln(Klotho)	1.433 (1.176,1.747)	<0.001	1.329 (1.077,1.641)	0.008
	Q1	Reference		Reference	
	Q2	1.059 (0.873,1.285)	0.562	1.076 (0.880,1.317)	0.476
	Q3	1.206 (0.998,1.460)	0.053	1.174 (0.962,1.434)	0.115
	Q4	1.390 (1.153,1.677)	0.001	1.327 (1.089,1.619)	0.005

fT4, free thyroxine; tT4, total thyroxine; fT3, free triiodothyronine; tT3, total triiodothyronine; TSH, thyroid-stimulating hormone; Q1,Q2,Q3,Q4, quartile grouping of ln(Klotho). The bold values indicate statistically significant differences.

Model 1(unadjusted) Covariates are not adjusted,Model 2(full-adjusted) Correct all covariates in the baseline table.

**Fig 3:**
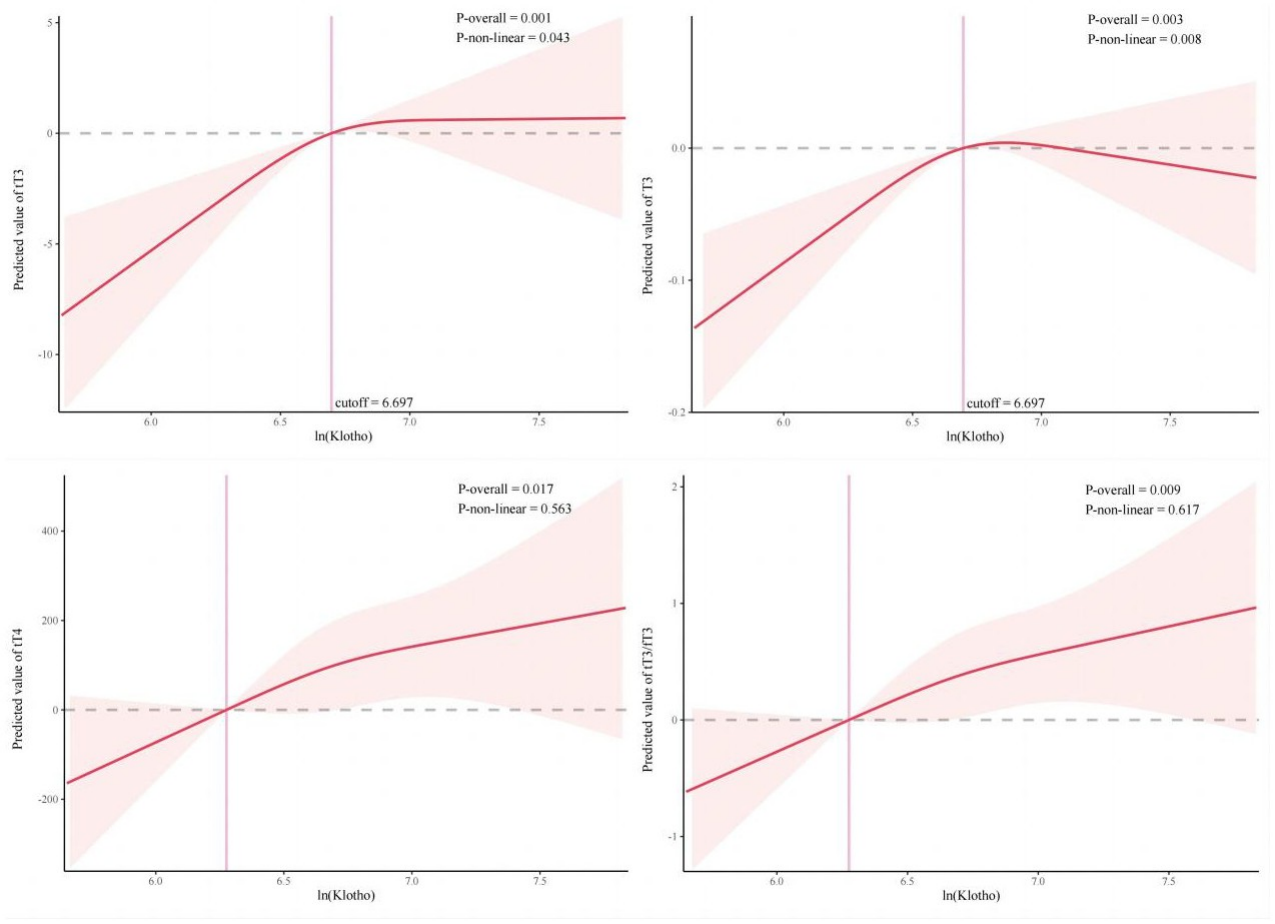
Restricted cubic spline plots between ln(Klotho) and tT3, fT3, tT4, tT3/fT3, with the number of knot nodes calculated as 4 according to the minimum AIC criterion,and the vertical line indicates the cutoff point. The model is full-adjusted (corrected for all covariates in the baseline).

Furthermore, to visualize the relationship between klotho proteins and thyroid hormones, a non-linearity test was first performed, and for variables with non-linear relationships, restriction cubic spline plots (RCS) were fitted, see Fig 3. Notably, we can find a non-linear relationship between klotho proteins and fT3 and tT3 (P-non-linear < 0.05). And the relationship between the two approximates an L-shape, with the cut-off point of 6.697 in both cases. When the cut-off point is exceeded, the relationship between klotho protein and fT3,tT3 gradually tends to be stable. However, the non-linear test between klotho protein and tT4 and tT3/fT3 was not significant (P-non-linear > 0.05), and the linear regression was sufficiently robust.

### Relationship between klotho protein and serum thyroid hormone in different strata

The stratification by gender showed a weaker association between ln(klotho) and fT3 in males compared with females (β = 0.051,95%CI:0.009,0.094) vs. (β = 0.056,95%CI:0.001,0.112). In addition, the associations between ln(klotho) and tT3, tT4, tT4/fT4, tT3/ fT3 were significant in males but not in females. The stratification by urinary iodine status showed a significantly positive association between ln(klotho) and tT3 in participants with urinary iodine >100 ug/L (β = 4.143,95%CI:1.312,6.975) and those with urinary iodine <100 ug/L (β = 4.262,95%CI:0.838,7.687), while the relationship with tT4, fT3, T4/fT4 and tT3/fT3 were significantly positively correlated only in participants with urinary iodine >100 ug/L (Table 4).

**Table 4:** Association between ln(Klotho) and thyroid hormones after sex and urinary iodine status subcomponent stratification.

	Male		Female		urinary iodine >100 ug/L		urinary iodine ≤ 100 ug/L	
	β (95% CI)	*P-value*	β (95% CI)	*P-value*	β (95% CI)	*P-value*	β (95% CI)	*P-value*
tT3	5.751 (2.975 to 8.528)	<0.001	2.642 (-0.792 to 6.076)	0.132	4.143 (1.312 to 6.975)	0.004	4.262 (0.838 to 7.687)	0.015
tT4	217.344 (6.830 to 427.857)	0.043	151.836 (-59.129 to 362.801)	0.158	293.122 (111.697 to 474.548)	0.002	-24.611 (-289.586 to 240.365)	0.855
fT3	0.051 (0.009 to 0.094)	0.001	0.056 (0.001 to 0.112)	0.046	0.063 (0.018 to 0.108)	0.006	0.040 (-0.012 to 0.093)	0.134
fT4	0.002 (-0.018 to 0.021)	0.860	0.007 (-0.015 to 0.028)	0.537	0.008 (-0.010 to 0.025)	0.396	0.003 (-0.023 to 0.028)	0.836
TSH	-0.154 (-0.425 to 0.118)	0.268	-0.220 (-0.715 to 0.276)	0.385	-0.320 (-0.698 to 0.057)	0.096	0.075 (-0.317 to 0.468)	0.706
tT4/tT3	-1.480 (-3.571 to 0.611)	0.165	0.071 (-2.313 to 2.454)	0.954	0.131 (-1.835 to 2.097)	0.896	-2.486 (-5.160 to 0.187)	0.068
fT4/fT3	-0.004 (-0.010 to 0.003)	0.242	-0.001 (-0.008 to 0.007)	0.838	-0.002 (-0.008 to 0.004)	0.509	-0.002 (-0.011 to 0.007)	0.682
tT4/fT4	249.560 (4.266 to 494.854)	0.046	67.224 (-177.067 to 311.514)	0.589	221.732 (16.012 to 427.452)	0.035	-46.462 (-371.686 to 278.762)	0.779
tT3/fT3	1.254 (0.525 to 1.982)	0.001	0.191 (-0.616 to 0.998)	0.643	0.671 (0.006 to 1.336)	0.048	0.850 (-0.111 to 1.812)	0.083

fT4, free thyroxine; tT4, total thyroxine; fT3, free triiodothyronine; tT3, total triiodothyronine; TSH, thyroid-stimulating hormone. The bold values indicate statistically significant differences.

## Discussion

In this large US population-based study, we found that tT3, tT4, fT3, and tT3/fT3 increased with klotho protein in US adults. ln(klotho) was significantly positively correlated with tT3, fT3, and significantly negatively correlated with TSH, tT4, fT4. After adjusting for covariates, ln(klotho) was still significantly correlated with tT3, fT3, tT4, and tT3/fT3 (P < 0.05). Through multivariate logistic regression, we found that klotho protein may be a risk factor for elevated fT3, tT3. And it is noteworthy that there is an approximately L-shaped nonlinear relationship between ln(klotho) and fT3, tT3 with a cut-off point of 6.697. Furthermore, the relationship was significant at values below the cut-off point and not significant at values above the cut-off points, suggesting the existence of threshold effects in the relationship between ln(klotho) and fT3, tT3.

The synthesis and breakdown of thyroid hormones are associated with the involvement of a variety of substances. Studies have proven that either an excess or deficiency of thyroid hormones can cause serious illness.For example, hypothyroidism is associated with coronary heart disease (CHD), dyslipidemia, atherosclerosis, renal insufficiency, and deficits in fine motor skills [18–22]. On the other hand, hyperthyroidism is linked to autoimmune diseases, heart failure, and atrial fibrillation [23,24]. Furthermore, several factors affect thyroid hormone levels, including genetics, exposure to pollutants, autoimmunity, inflammation, and oxidative stress [25]. Recent study have identified a possible association between klotho proteins and thyroid hormones [26], but the exact relationship has not been proven.

Klotho, an anti-aging gene, also has anti-inflammatory and anti-oxidative stress effects. In humans, the serum levels of klotho proteins decrease with age from the age of 40. Klotho proteins are highly expressed in the kidneys, cerebral choroid plexus, parathyroid glands, pituitary gland, thyroid gland, aorta, ovaries, and testes [27–29]. Klotho protein is closely associated with various diseases. Elderly patients with hypertension show significantly lower serum klotho levels than those without hypertension, suggesting that the development of hypertension in the elderly population could be associated with reduced levels of klotho protein [30–32]. Furthermore, patients with chronic renal failure show significantly reduced levels of klotho proteins in the renal tissue, which could be explained by the fact that decreased expression of exacerbation exacerbates interstitial fibrosis [33]. In addition, decreased expression of the klotho protein is involved in the development of chronic obstructive pulmonary disease and Alzheimer’s disease [34]. Furthermore, klotho over-expression was shown to significantly reduce follicular thyroid cancer FTC133 and FTC238 cells proliferation and enhance apoptosis [35]. Qiong Wu et al. demonstrated that klotho inhibited cell proliferation in a RET fusion model of PTC by inhibiting the Wnt/β-linked protein pathway [36]. The anti-inflammatory and anti-oxidative stress effects of klotho protein could affect thyroid hormones. However, no studies have investigated the correlation between serum klotho levels and thyroid hormones.

In our study, we found that klotho protein may be a risk factor for elevated tT3, fT3, and the results showed that there was a threshold effect in the relationship between ln(klotho) and fT3, tT3. More notably, the cut-off points were both 6.697. By calculation, we found that the mean fT3 value corresponding to the cut-off point was 116.5 ng/dL and the mean tT3 value was 3.1325 nf/dL, which were both within the normal range. We speculate that when ln(klotho) < 6.697, the trend of fT3 and tT3 increasing with it is significant, while when ln(klotho) is greater than 6.697, the trend of fT3 and tT3 increasing with it is no longer obvious, and the specific reasons need to be verified by further mechanism exploration.

This study showed gender disparities in the relationship between serum klotho levels and thyroid hormones, with males having a lower association between ln(klotho) and fT3 than females. Furthermore, the associations between ln(klotho) and tT3, tT4, tT4/fT4, and tT3/fT3 were significant in males but not in females. Previous studies have shown that estrogen increases the expression of thyroglobulin, thyroid peroxidase activity, and iodine uptake, and regulates TSH levels [37–40]. In addition, estrogen modifies the activity and expression of dual oxidase 2 (DUOX2) and nicotinamide adenine dinucleotide phosphate oxidase 4 (NOX4), thus influencing the thyroid redox state [41]. Therefore, klotho protein may have a considerably lower effect on thyroid hormones than estrogen, indicating that male adults may show more effects of Klotho protein on thyroid hormones than females. However, further studies are needed to show the potential pathways behind the gender disparities between serum klotho protein levels and thyroid hormones.

In addition, previous studies have shown that iodine deficiency and iodine overload could cause thyroid dysfunction since iodine is a micronutrient necessary for the synthesis of thyroid hormones. The present study showed a significantly positive correlation between ln(klotho) and tT3, fT3, tT4/fT4, and tT3/fT3 in participants with urinary iodine >100 ug/L. In contrast, ln(klotho) was significantly positively correlated with tT3 in participants with urinary iodine <100 ug/L. This phenomenon could be explained by the fact that iodine deficiency causes a decrease in circulating TT3 and TT4, resulting in increased TSH release from the pituitary gland through a classical negative feedback mechanism [42], which could diminish the effects of klotho protein on thyroid hormones.

This study had some limitations. First, as this paper is a cross-sectional study, causality cannot be inferred and future longitudinal studies are needed to determine the predictive value of Klotho in thyroid disorders. Secondly, most of the data were obtained from single-site measurements, and without data on concentration changes, our results can only reflect the relationship between klotho and thyroid hormones at the initial stage of the study. Finally, despite our attempts to adjust for potential confounders as much as possible, there remain a number of unknown or unmeasured confounders that may also play a role in the pathogenesis of thyroid disease. Therefore, multiple measurements in a long-term follow-up study would likely show the association between serum klotho protein levels and thyroid hormones.

In conclusion, the present study demonstrated a correlation between klotho protein levels and thyroid function in U.S. adults. Furthermore, the relationship between klotho protein levels and thyroid hormones is more pronounced in men and adults with adequate iodine levels. However, large prospective studies are required to generalize the findings of this study.

## Conclusion

Serum klotho levels are associated with tT3, tT4, fT3, TSH, tT4/fT4, tT3/fT3, tT4/tT3, and fT4/fT3, and klotho protein may be a risk factor for elevated fT3,tT3. We suggest that klotho could be involved in the physiological regulation of thyroid function.

## Abbreviations

CHD: Coronary heart disease
CRYM: μ-Crystallin
DIT: Diiodotyrosine
DUOX2: Dual oxidase 2
MIT: Monoiodotyrosine
NCHS: National Center for Health Statistics
NHANES: National Health and Nutrition Examination Survey
NIS: Sodium/iodide isotransporter protein
NOX4: Nicotinamide adenine dinucleotide phosphate oxidase 4
RCS: Restricted cubic spline plots
T3: Triiodothyronine
T4: Thyroxine
TG: Thyroglobulin
TPO: Thyroid peroxidase
TSH: Thyroid stimulating hormone
